# Supplementary material for: Microbial Community Analyses Associated with Nine Varieties of Wine Grape Carposphere Based on High-Throughput Sequencing
Source: Microorganisms. 2019 Dec 9;7(12):668. doi: 10.3390/microorganisms7120668 (PMC6956142; doi:10.3390/microorganisms7120668)
Supplement: Supplementary file 1 [file microorganisms-07-00668-s001.zip › microorganisms-638829-supplementary/Supplementary/Supplementary-table.docx]

**Table S1** Core bacterial OTUs and most similar species on the grape samples

|  | Reference species (RS) | accession no. | Identity（%） |
| --- | --- | --- | --- |
| OTU1 | *Pseudomonas* sp*.* | KM187516.1 | 100 |
| OTU2 | *Bacillus* sp*.* | KT001003.1 | 99 |
| OTU3 | *Vagococcus* sp*.* | KT8860405.1 | 99 |
| OTU4 | *Arthrobacter* sp*.* | GU377096.1 | 98 |
| OTU5 | *Planococcus* sp*.* | HQ327129.1 | 99 |
| OTU6 | *Pantoea* sp*.* | KP099965.1 | 99 |
| OTU7 | *Pseudarthrobacter* | MG753544.1 | 99 |
| OTU8 | *Curtobacterium* sp*.* | KJ 184990.1 | 100 |
| OTU9 | *Arthrobacter* sp*.* | KU060866.1 | 95 |
| OTU10 | *Pseudomonas* sp*.* | KT583556.1 | 97 |
| OTU11 | *Cellulomonas* sp*.* | KM507609.1 | 99 |
| OTU12 | *Caulobacter* sp*.* | JQ723715.1 | 99 |
| OTU13 | *Corynebacterium* sp. | KU041664.1 | 99 |
| OTU14 | *Stenotroophomonas* sp*.* | KT274784.1 | 99 |
| OTU15 | *Massilia* sp*.* | KR922198.1 | 99 |
| OTU16 | Uncultured *Gemmatimonas* sp*.* | HE974816.1 | 100 |
| OTU17 | *Massilia* sp*.* | FR865952.1 | 99 |
| OTU18 | *Fillbacter* sp*.* | KF313425.1 | 100 |
| OTU19 | uncultured bacterium | JQ825178.1 | 96 |
| OTU20 | *Microvirga* sp*.* | KM598263.1 | 100 |
| OTU21 | *Dietzia* sp*.* | KP722602.2 | 99 |

**Table S2** Core fungal OTUs and most similar species on the grape samples

|  | Reference species (RS) | accession no | Identity（%） |
| --- | --- | --- | --- |
| OTU1 | *Alternaria* sp. | KT269179.1 | 100 |
| OTU2 | *Cladosporium* sp. | LN808877.1 | 100 |
| OTU3 | *Phoma* sp. | JN207353.1 | 99 |
| OTU4 | *Alternaria* sp. | MH567001.1 | 100 |
| OTU5 | *Cladosporium* sp. | KF367491.1 | 100 |
| OTU6 | *Davidiella* sp. | JX164064.1 | 100 |
| OTU7 | *Phoma* sp. | KF367492.1 | 100 |
| OTU8 | *Fusarium* sp. | KJ472204.1 | 100 |
| OTU9 | *Cladosporium* sp. | LN808858.1 | 100 |
| OTU10 | uncultured fungus | HG935338.1 | 99 |
| OTU11 | uncultured fungus | HG935374.1 | 99 |
| OTU12 | *Rhodotorula* sp. | AM901704.1 | 100 |
| OTU13 | *Cryptococcus* sp. | JX164067.1 | 100 |
| OTU14 | *Fusarium* sp. | KT268430.1 | 100 |
| OTU15 | *Epicoccum* sp. | JX014389.1 | 100 |
| OTU16 | uncultured fungus | JX390488.1 | 100 |
| OTU17 | *Cryptococcus* sp. | AM901689.1 | 100 |
| OTU18 | uncultured fungus | KU931455.1 | 100 |
| OTU19 | uncultured fungus | JX345540.1 | 100 |
| OTU26 | *Cylindrocarpon* sp. | KM248582.1 | 100 |
| OTU30 | *Cryptococcus* sp. | JN255470.1 | 100 |
| OTU43 | uncultured zygomycete | FJ777142.1 | 97 |
| OTU47 | *Cryptococcus* sp. | JX164069.1 | 100 |
| OTU58 | *Sebacinaceae* sp. | JX138554.1 | 91 |
| OTU59 | uncultured fungus | JX389839.1 | 97 |
| OTU60 | *Basiomycete* sp. | EU098119.1 | 98 |
| OTU77 | uncultured fungus | JX378973.1 | 99 |
| OTU79 | uncultured Sordaria | HG937029.1 | 99 |
